# The mirid bug *Apolygus lucorum* deploys a glutathione peroxidase as a candidate effector to enhance plant susceptibility

**DOI:** 10.1093/jxb/eraa015

**Published:** 2020-01-17

**Authors:** Yumei Dong, Maofeng Jing, Danyu Shen, Chenyang Wang, Meiqian Zhang, Dong Liang, Karani T Nyawira, Qingyue Xia, Kairan Zuo, Shuwen Wu, Yidong Wu, Daolong Dou, Ai Xia

**Affiliations:** 1 College of Plant Protection, Nanjing Agricultural University, Nanjing, China; 2 The James Hutton Institute, UK

**Keywords:** *Apolygus lucorum*, cell death, effector, glutathione peroxidase, plant–insect interaction, ROS

## Abstract

The mirid bug *Apolygus lucorum* has become a major agricultural pest since the large-scale cultivation of Bt-cotton. It was assumed that *A. lucorum*, similarly to other phloem sap insects, could secrete saliva that contains effector proteins into plant interfaces to perturb host cellular processes during feeding. However, the secreted effectors of *A. lucorum* are still uncharacterized and unstudied. In this study, 1878 putative secreted proteins were identified from the transcriptome of *A. lucorum*, which either had homology with published aphid effectors or shared common features with plant pathogens and insect effectors. One hundred and seventy-two candidate effectors were used for cell death-inducing/suppressing assays, and a putative salivary gland effector, *Apolygus lucorum* cell death inhibitor 6 (Al6), was characterized. The mRNAs of Al6 were enriched at feeding stages (nymph and adult) and, in particular, in salivary glands. Moreover, we revealed that the secreted Al6 encoded an active glutathione peroxidase that reduced reactive oxygen species (ROS) accumulation induced by INF1 or Flg22. Expression of the Al6 gene *in planta* altered insect feeding behavior and promoted plant pathogen infections. Inhibition of cell death and enhanced plant susceptibility to insect and pathogens are dependent on glutathione peroxidase activity of Al6. Thus, this study shows that a candidate salivary gland effector, Al6, functions as a glutathione peroxidase and suppresses ROS induced by pathogen-associated molecular pattern to inhibit pattern-triggered immunity (PTI)-induced cell death. The identification and molecular mechanism analysis of the Al6 candidate effector in *A. lucorum* will provide new insight into the molecular mechanisms of insect–plant interactions.

## Teaser

A candidate effector with glutathione peroxidase activity from the *Apolygus lucorum* salivary gland suppressed PAMP-triggered cell death and ROS accumulation to enhance plant susceptibility to insects and pathogens.

## Introduction

Sap-sucking insects such as aphids, planthoppers, whiteflies, and other Hemiptera insert their needle-like stylets into the plant epidermis, puncture the phloem tissue, and feed from the nutrient-rich sap (Kettles and Kaloshian, 2016). During this probing, ‘non-self’ molecules belonging to the invading insect such as herbivore-associated molecular patterns (HAMPs) or damage-associated molecular patterns (DAMPs) are recognized by plant pattern recognition receptors (PRRs) (Guiguet *et al.*, 2016). Insect perception by plants triggers complex defense responses, known as pattern-triggered immunity (PTI), which includes the burst of reactive oxygen species (ROS), calcium signaling, deposition of callose, activation of mitogen-activated protein kinases (MAPKs), and hormone signaling mediated by jasmonic acid (JA), salicylic acid (SA), and ethylene (kmm ) (Acevedo *et al.*, 2015; Bigeard *et al.*, 2015). To enable successful feeding and infestation, insects deliver a series of effectors from salivary glands into their host cells to suppress PTI and modulate herbivore–plant interactions (Hogenhout and Bos, 2011). However, with time, some plants may have adapted to carry *R* (resistance) genes that can recognize these effectors to mount a resistance response, called effector-triggered immunity (ETI) (Stuart, 2015). The arms race continues with specialist herbivores by exploring effectors to evade detection or suppress ETI (Bruce, 2015). Over millions of years of co-evolution, phloem feeders have developed dynamic and complex interactions with plant hosts.

Identification of insect effectors and understanding their role in modulating plant defenses may provide valuable information for the development of novel pest management strategies. Over the past decade, available literature on sap feeding and chewing insect effectors has revealed exciting insight into the molecular determinants of plant–insect interactions (Hogenhout and Bos, 2011; Bruce, 2015). The first effector identified in the saliva of herbivores is glucose oxidase (GOX) from a caterpillar (*Helicoverpa zea*) with the ability to suppress the JA defense signaling pathway (Musser *et al.*, 2002). In the following years, a number of salivary gland effectors have been characterized from aphid, Hessian fly, whitefly, cotton bollworm, and planthopper (Rodriguez *et al.*, 2017; Shangguan *et al.*, 2018). Previous data revealed that MP10 from the aphid species *Myzus persicae* and a mucin-like protein of planthopper act as elicitors by inducing cell death and triggering defense responses in plants (Bos *et al.*, 2010; Shangguan *et al.*, 2018). Additionally, a macrophage migration inhibitory factor (MIF) secreted in aphid (*Acyrthosiphon pisum*) saliva inhibits plant immune responses (Naessens *et al.*, 2015). The brown planthopper *Nilaparvata lugens* NlSEF1 (a salivary EF-hand calcium binding) protein regulates the levels of Ca^2+^ and H_2_O_2_, but not JA, jasmonoyl-isoleucine (JA-Ile), and SA, in rice (Ye *et al.*, 2017). An effector named HARP1 from cotton bollworm (*Helicoverpa armigera*) oral secretion blocked JA signaling transduction in Arabidopsis through interactions with JASMONATE-ZIM-DOMAIN (JAZ) repressors to prevent COI1-mediated JAZ degradation (Chen *et al.*, 2019). Overall, most known salivary gland effectors were found to modulate plant defenses by targeting calcium signaling, hormone pathway, or others, but only one effector, BtFer1 from whitefly *Bemisia tabaci*, has been functionally analyzed to manipulate the host ROS pathway (Su *et al.*, 2019).

Glutathione peroxidases (GPxs) are a family of phylogenetically related enzymes that use glutathione (GSH) as an electron to catalyze the conversion of H_2_O_2_ or organic hydroperoxides to water or alcohols (Margis *et al.*, 2008). GPxs exert diverse biological roles in developmental processes and defenses against stresses and pathogen infections (Brigelius-Flohé and Maiorino, 2013). In plants, the multiple roles of GPxs in stress responses have been extensively studied, and the molecular mechanisms of GPxs as antioxidant enzymes have been revealed either by eliminating reactive oxygen species (ROS) to maintain H_2_O_2_ homeostasis, or by participating in protein complexes or signaling pathways involved in stress defense (Bela *et al.*, 2015). Plant GPxs are also associated with cell defense against pathogen attack; for instance, Hessian fly feeding induces increased levels of GPx activity in wheat plants (Liu *et al.*, 2015). However, the mechanisms of GPxs in response to pathogens are poorly understood. In insects, available evidence suggests that GPxs in combination with other antioxidant enzymes are implicated in insect growth, development, and aging by maintaining endogenous oxidative homeostasis (Ahmad and Pardini, 1990). Interestingly, several potential effectors with the GPx domain have been predicted to be present in the salivary glands of Hessian fly (*Mayetiola destructor*), pea aphid (*Acyrthosiphon pisum*), and potato aphid (*Macrosiphum euphorbiae*) (Chen *et al.*, 2008; Carolan *et al.*, 2011; Atamian *et al.*, 2013). However, the molecular roles of GPxs in host defense manipulation are still unexplored.

*Apolygus lucorum* (Meyer-Dur) (Heteroptera: *Miridae*), with wide distribution in Asia, Europe, Africa, and Northern America, has become one of the most important agricultural pests, especially in China (Li *et al.*, 2017; Zhang *et al.*, 2017). Since the late 1990s, due to the large-scale cultivation of genetically modified Bt (*Bacillus thuringiensis*) cotton, *A. lucorum* has replaced lepidopteron species as a primary pest in the cotton fields (L. Zhang *et al.*, 2015). As a polyphagous species, *A. lucorum* gradually migrated to a wide range of plants including many important crops and fruit trees (Tan *et al.*, 2016). Therefore, it has caused substantial economic losses to agricultural crops in China. Like other piercing–sucking insects, this mirid bug secretes a series of salivary enzymes into plants when sucking to interfere with host immune responses and benefit bug feeding (Zhang *et al.*, 2017). Several digestive enzymes aiding in the digestion of food, including pectinase, amylase, cellulase, and protease, were identified from the saliva of *A. lucorum*, and enzyme activities were also determined (Tan *et al.*, 2016; Li *et al.*, 2017). The functions of two polygalacturonase enzymes in *A. lucorum* were demonstrated using RNAi; these enzymes were able to elicit plant injury after injection into plant cells (L. Zhang *et al.*, 2017). However, no salivary effectors that modulate host defense responses have been studied in *A. lucorum* to date.

In this study, we combined transcriptome investigation and aphid salivary gland effector analysis to identify candidate effectors in *A. lucorum*. Using *Agrobacerium tumefaciens* infiltration assays, an *A. lucorum* candidate effector 6, named Al6, was characterized to inhibit pathogen-associated molecular pattern (PAMP)-triggered cell death. Molecular functional analysis demonstrated that Al6 acted as a GPx to inhibit PAMP-induced ROS for suppressing the plant defense response. Transient *in planta* expression of Al6 altered insect feeding behavior and pathogen resistance.

## Materials and methods

### Insects and plant materials

*Apolygus lucorum* and *H. armigera* Hubner were routinely stored in the insectary room. *Apolygus lucorum* was maintained at 25±1 °C and 55±5% relative humidity, with a 14:10 h (light:dark) photoperiod. Larvae of *A. lucorum* were fed with green pods and corn, and adults were provided with 10% sucrose solution. *Helicoverpa armigera* was kept at 25±1 °C with a 14:10 (light:dark) photoperiod, and larvae were reared on an artificial diet made from wheat germ and soybean powder. Adults were supplied with a 10% sugar solution. *Nicotiana benthamiana* was kept at 25 °C and 60% relative humidity under a 16/8 h (light:dark) photoperiod.

### Bioinformatics analysis

Total RNA from whole bodies of *A. lucorum* was extracted using the RNA simple Total RNA Kit (Tiangen, China) according to the manufacturer’s instructions, and then sequenced with the Illumina NGS platform to generate high-throughput RNA sequencing (RNA-Seq) data. The resultant raw reads were processed by removing poor quality reads and trimming adaptors. In the absence of a reference genome of *A. lucorum*, the clean reads were imported into the Trinity assembler with the default parameters for *de novo* assembly (Grabherr *et al.*, 2011). For the assembled transcripts, the TransDecoder program was used to identify candidate coding regions. Signal peptides were predicted using SignalP v3.0 (Bendtsen *et al.*, 2004). The aphid effector sequences were retrieved from publications (Bos *et al.*, 2010; Atamian *et al.*, 2013), and used as queries for Blast searches (E-value <1×10^–5^) against *A. lucorum* secreted proteins. The domain component in each protein sequence was predicted using the Pfam database (Finn *et al.*, 2016). To predict GPx domain-containing proteins in each insect, the hidden Markov model profile of GPx (PF00255) was obtained from the Pfam database, and then used to perform a HMM search against insect proteins using the HMMER program (Finn *et al.*, 2011).

### *Agrobacterium tumefaciens* infiltration assays

The candidate effector cDNAs were amplified from isolated total RNA of *A. lucorum*, and the signal peptides were removed for insertion into the pBinGFP2 vector under the control of the 35S promoter (Song *et al.*, 2015). The successful constructed plasmids were then transformed into *A. tumefaciens* strain GV3101 by electroporation (Olivier *et al.*, 2003). Successful transformants were confirmed with PCR amplification using Pbin-AI6 primers (Supplementary Table S1 at *JXB* online). Recombinant strains of *A. tumefaciens* were cultured, washed, and re-suspended in infiltration buffer (10 mM MgCl_2_, 500 mM MES, 100 mM acetosyringone) until an appropriate optical density (OD) of 0.4 at 600 nm was reached to harvest for infiltration. *Nicotiana benthamiana* leaves that were 4–6 weeks old were used to conduct infiltration assays using a needleless syringe (Olivier *et al.*, 2003). For the induction of cell death assays, a well-known PAMP from the plant pathogen *Phytophthora infestans*, INF1, was used as a positive control, while empty vector (EV) was the negative control. To evaluate the effect of *A. lucorum* effectors on INF1-induced cell death, *N. benthamiana* leaves were first infiltrated with recombinant strains of *A. tumefaciens* carrying candidate effector genes or green fluorescent protein (GFP), and INF1 was injected in the same region after 12 h. BAX is a mouse apoptosis-associated protein that was used to test the inhibitory effect of AI6. BAX was injected 24 h after AI6 or GFP infiltration. Symptom development of the injected leaves was photo-recorded.

### Western blots

Agroinfiltrated leaves cultivated for 48 hours post-infiltration (hpi) were harvested for protein extraction. Proteins were prepared according to a previously described method (M. Zhang *et al.*, 2015). After electrophoresis, proteins were transferred from the gel to a membrane for immunoblotting using mouse anti-HA monoclonal antibodies (Sigma-Aldrich) and goat anti-mouse IRDye 800CW [Odyssey (no. 926-32210); Li-Cor]. The membrane was finally washed and visualized using Odyssey with excitation at 700 nm and 800 nm (M. Zhang *et al.*, 2015).

### Developmental stage- and tissue-specific expression patterns of Al6

Different developmental stages of *A. lucorum* including first, second, third, fourth, and fifth instar nymphs, and adults were used to detect the relative transcriptional expression of Al6. Salivary gland, head, thorax, abdomen, leg, and wing were dissected from *A. lucorum* adults. Total RNA was isolated using the RNA simple Total RNA Kit (Tiangen, China). Quantitative real-time PCR (qRT-PCR) was performed using Al6RT-F and Al6RT-R primers as previously described (Wang *et al.*, 2019), and the primers used in this experiment are shown in Supplementary Table S1. The relative expression levels of the Al6 gene were normalized using the housekeeping genes β-actin and GAPDH as internal standards. At least three biologically independent replicates were carried out for each sample.

### ROS assays

For testing the ROS burst in the leaves by DAB (3,3′-diaminobenzidine) staining, detached leaf samples were collected at 2 d after infiltration of *A. tumefaciens* strains as previously described (Dong *et al.*, 2011). Leaves were stained with 1 mg ml^–1^ DAB solution (Sigma-Aldrich, USA) at 25 °C for 6 h, then decolorized with boiling alcohol, and kept in 30% glycerin. To determine the role of Al6 in the ROS burst in response to flg22, a luminol-based assay was adopted as previously described (Trujillo, 2016). After the instantaneous expression of GFP and Al6 in *N. benthamiana* for 36 h, leaf disks were washed with water and kept overnight in the dark at room temperature. Then, the leaf disks were stained in test buffer containing luminol (Sigma-Aldrich, USA), horseradish peroxidase (Sigma-Aldrich, USA), and 1 μM flg22 (GenScript, China), and luminescence detection was conducted for at least 30 min in the microplate reader (BioTek, China).

### Glutathione peroxidase activity assay and mutant construction

The coding region of Al6 without signal peptide was amplified with the pET-Al6F and pET-Al6R primers (Supplementary Table S1), and cloned into the PET-32a expression vector with a His tag. Recombinant PET-32a vector was transformed into *Escherichia coli* strain BL21 with 0.1 mM isopropyl-β-d-thiogalactopyranoside (IPTG) by incubating at 37 °C for 6 h. The protein expression level was determined by Coomassie brilliant blue staining and western blot. GPx activity was measured using a Cellular Glutathione Peroxidase Assay Kit (Beyotime, China) with PET-32a EV as a negative control. Three point mutants of Al6 were constructed using overlap PCR with specific primers: CA-F1c, CA-R1b, WA-F3c, and WA-R3b (Supplementary Table S1). The amplified fragments were inserted into a plant expression vector (pBinGFP2) and a prokaryotic expression vector (PET-32a).

### Insect feeding and pathogen infection assays

After the instantaneous expression of GFP and Al6 in *N. benthamiana* for 48 h, eight second instar larvae of *H. armigera* were placed in the middle of these leaves, which were photo-recorded after treatment at 24 h and 48 h. All analyses were repeated at least three times. The *Phytophthora parasitica* strain PP025 used in this study was cultured at 25 °C in the dark on 10% (v/v) V8 juice medium, and *Sclerotinia sclerotiorum* was cultured at 25 °C in the dark on PDA medium. For mycelium inoculation on *N. benthamiana*, equal amounts of growth medium were inoculated on one half of the leaf 24 hpi. The *P. parasitica*-inoculated leaves were photographed under UV light at 36 and 48 hpi, and the lesion length was measured. The *S. sclerotiorum*-inoculated leaves were photographed under white light at 72 hpi. This assay was repeated at least three times.

### Accession numbers

The sequence of Al6 was submitted to the NCBI GenBank under the accession number MN149616.

## Results

### Identification of *A. lucorum* salivary gland candidate effectors

**Fig. 1.**
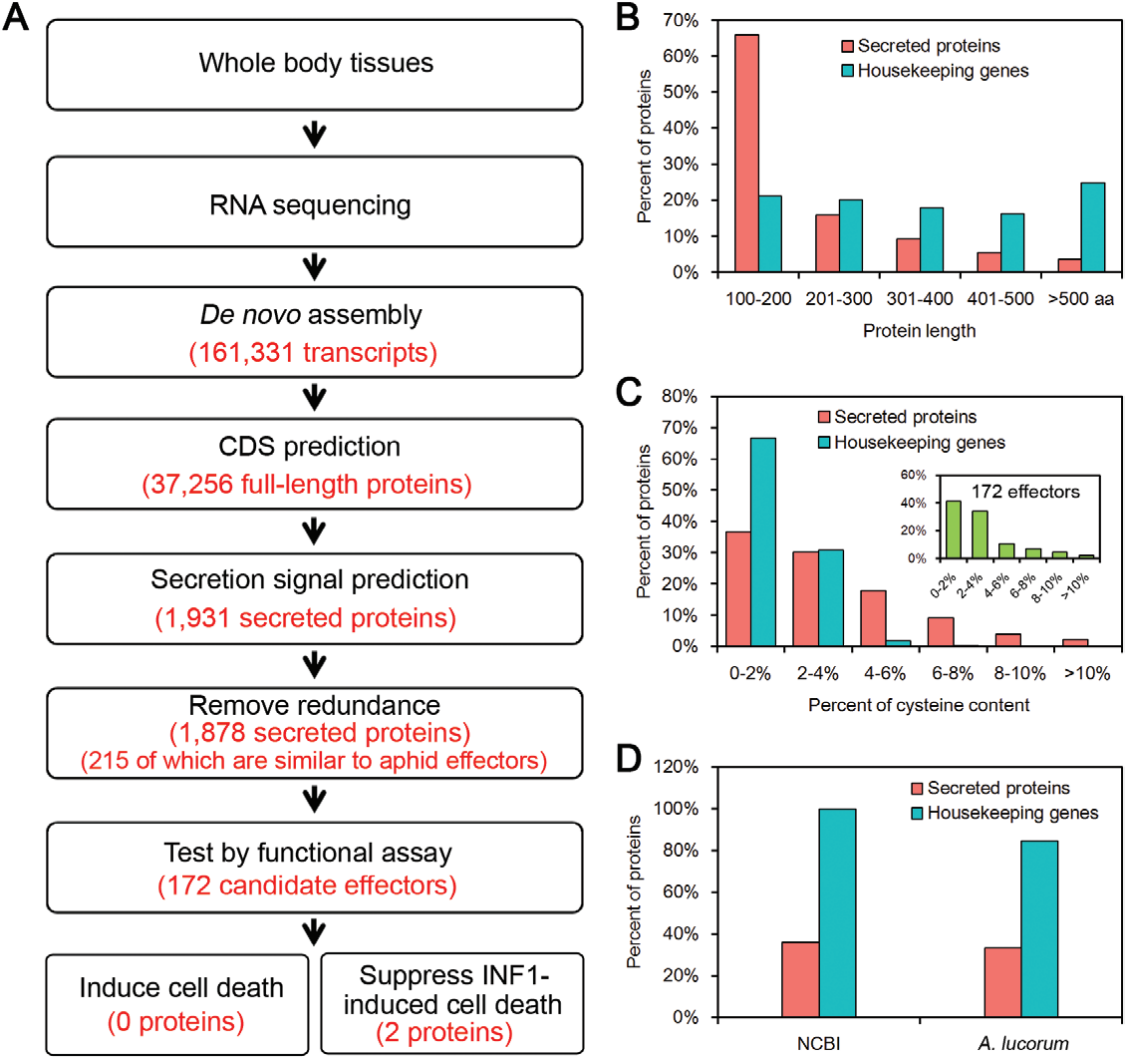
Identification of *Apolygus lucorum* candidate effector proteins. (A) Bioinformatics pipeline for the identification of candidate effectors from *A. lucorum*. (B) The protein length distribution of *A. lucorum* secreted proteins compared with housekeeping genes. (C) The cysteine content distribution of *A. lucorum* secreted proteins compared with housekeeping genes. (D) Comparison of NCBI homolog and *A. lucorum* paralog numbers between *A. lucorum* secreted proteins and housekeeping genes. (This figure is available in color at *JXB* online.)

To identify *A. lucorum* candidate effectors from salivary glands, a library constructed using RNA extracted from the whole body of *A. lucorum* was sequenced with the Illumina NGS platform to generate high-throughput RNA-Seq data. By *de novo* assembly, a total of 161 331 transcripts were obtained (Fig. 1A). *Apolygus lucorum* effectors were hypothesized to be secreted proteins that are delivered into the watery saliva. The SignalP v3.0 program was used to characterize secreted proteins with the feature of an N-terminal signal peptide. Therefore, 37 256 full-length ORFs were identified in the assembled transcripts, and 2198 ORFs among these were predicted to contain signal peptides. Among these proteins with signal peptides, 267 predicted proteins with transmembrane domains were ruled out considering that they were likely to be anchored to the membranes. In addition, 53 redundant proteins sharing >95% sequence similarity were also removed. Hence, 1878 predicted secreted proteins remained for subsequent analysis. Among the piercing–sucking insects, the aphid is the best model insect in the study of salivary gland effectors (Rodriguez *et al.*, 2013). It belongs to the same order of Hemiptera as *A. lucorum*, but the aphid is a member of the *Aphidoidea* family and *A. lucorum* is within the family of *Miridge* (Peccoud *et al.*, 2010; Tan *et al.*, 2016). So, the effector sequences of pea aphid (*Acyrthosiphon pisum*) were used as a reference to evaluate the accuracy of predicted effectors in *A. lucorum* (Carolan *et al.*, 2011). The result showed that 215 secreted proteins of *A. lucorum* are highly conserved in *A. pisum* (Fig. 1A), indicating that our prediction is reliable.

Studies on plant pathogens and insects revealed some common features of effector proteins such as short amino acid sequences, cysteine-rich residues, or high sequence diversity (Hogenhout and Bos, 2011; Dou and Zhou, 2012). We further analyzed 1878 secreted proteins, and the results showed that 81.9% of secreted proteins had a short protein length of fewer than 300 amino acids, compared with only 41.2% in the *A. lucorum* housekeeping genes (Fig. 1B). Of them, 15.3% of secreted proteins contained cysteine-rich residues (>4%), in contrast to only 0.6% in the housekeeping genes (Fig. 1C). Based on Blast analysis (E-value <1×10^–5^), 35.9% and 33.2% of secreted proteins shared sequence similarity with the NCBI NR database and *A. lucorum* proteins, respectively, whereas the majority of housekeeping genes had homologs in the two data sets using same Blast search criteria (Fig. 1D). Taken together, 1878 of *A. lucorum* secreted proteins can be considered potential salivary gland effectors.

### Screening of *A. lucorum* candidate effector 6

**Fig. 2.**
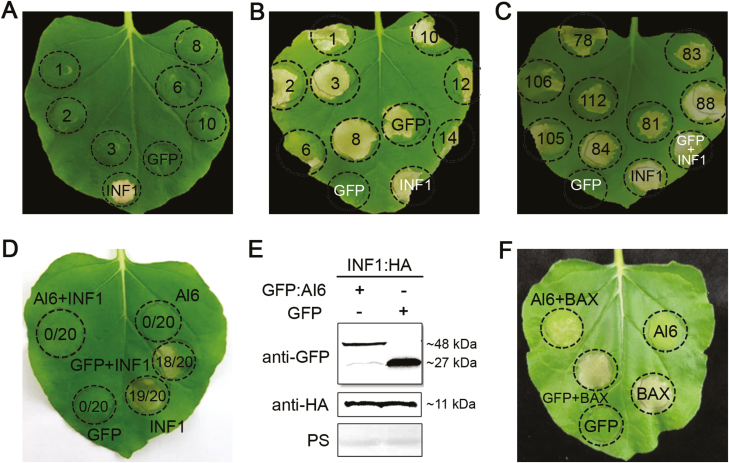
Screening of *A. lucorum* candidate effectors. (A) *Agrobacterium tumefaciens* infiltration assay for screening candidate effectors inducing cell death. Candidate effectors, the cell death-inducing gene *INF1*, and empty control (*GFP*) were expressed in *Nicotiana benthamiana* leaves via agroinfiltration. GFP is used as the negative control, and INF1 is the positive control that induced cell death. (B and C) *A. tumefaciens* infiltration assay for screening effectors inhibiting cell death. *N. benthamiana* leaves were first infiltrated with recombinant strains of *A. tumefaciens* carrying candidate effector genes or *GFP*, and *INF1* was injected in the same region after 12 h. The numbers in the circle indicate the candidate effector numbers. The leaves were photographed 7 d after agroinfiltration and three biological repeats were performed for each candidate effector. (D) The leaves of *N. benthamiana* in which INF1-triggered cell death was inhibited by the expression of *Al6*. GFP is the negative control that cannot induce cell death. The ratios in the circle represent the time of cell death relative to the total time of the experiments. The photographs were taken at 7 d post-infiltration. (E) Western blot detection of the Al6 and INF1 proteins. Anti-GFP and anti-HA antibodies were used to detect the expression of the indicated constructs, and equal loading of each sample is indicated by Ponceau staining of Rubisco protein (PS). (F) Al6 inhibits BAX-induced cell death. (This figure is available in color at *JXB* online.)

In plant pathology, the most common feature of effector proteins is to regulate host cell processes through cell death induction or PTI suppression (Dou and Zhou, 2012). To investigate the functions of *A. lucorum* predicted effectors in plant, a total of 172 candidate effectors were randomly selected from 1878 secreted proteins for transient expression in the leaves of *N. benthamiana* with agroinfiltration, and then cell death induction or suppression assays were performed. In the cell death induction assay, GFP was used as a negative control, and PAMP INF1, which is the most commonly used cell death inducer in plant immunity, was used as a positive control. After infiltration of candidate effectors into *N. benthamiana* for 2 d, cell death induced by INF1 was observed at the infiltration site, but no cell death phenotype was observed for 172 candidate effectors or GFP, even after 10 d of infiltration (Fig. 2A). This result suggested that no candidate effector causing host cell death was identified from our data mining.

Candidate effectors inhibiting INF1-induced cell death were also investigated by infiltration of INF1 into the expressed candidate effectors or GFP for 24 h. Cell death symptoms appeared at the injection site upon overexpression of INF1 on the third day for all samples. After infiltration of the candidate effectors, only candidate protein 6 and 106 from 172 candidate effectors showed inhibition of cell death induced by INF1 (Fig. 2B, C). To further confirm this result, we repeated the experiments 20 times and the data confirmed that PAMP INF1-triggered cell death in *N. benthamiana* was suppressed by the overexpression of Al6 (Fig. 2D) and Al106 (image not shown). Western blot analysis was used to confirm the expression of Al6 and INF1 in *N. benthamiana* (Fig. 2E). The result revealed that the expression of Al6 did not have any effect on the protein level of INF1, suggesting that Al6 did not alter the accumulation of INF1, but might interfere in downstream pathways triggered by INF1. We further tested whether Al6 may inhibit different types of cell death in *N. benthamiana*, and another most commonly used pro-apoptotic elicitor inducing ETI-triggered cell death in plants, BAX from mouse, was used to conduct the inhibitory cell death experiment. Our data demonstrated that Al6 also suppressed BAX-induced cell death in *N. benthamiana* (Fig. 2F). Taken together, our results conclude that *A. lucorum* candidate effector 6, named Al6, acts as an inhibitor of plant cell death.

### Al6 is highly expressed in the salivary gland

**Fig. 3.**
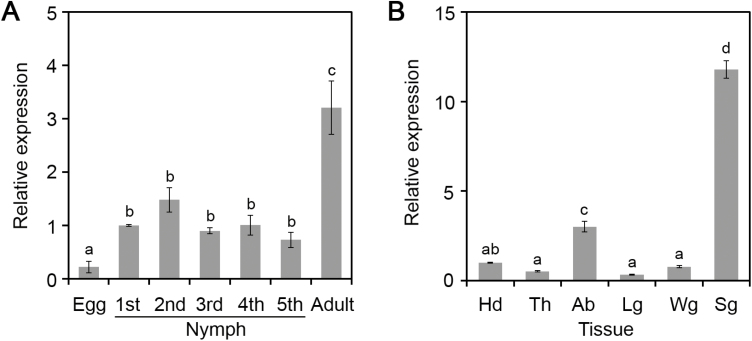
Al6 is highly expressed in the salivary glands. (A) Relative expression levels of *Al6* at different development stages (egg, first to fifth instar, and adult). Data were normalized against β-actin gene expression, and the bars represent means (±SD) of three repeats. Different letters above the bars indicate significant differences, as determined by Tukey’s honestly significant difference test (*P<0.05). (B) Relative expression patterns of *Al6* in different tissues (Sg, salivary gland; Hd, head; Th, thorax; Ab, abdomen; Lg, leg; Wg, wing).

To test that Al6 is specifically expressed in salivary glands, we analyzed the relative expression levels of *Al6* in *A. lucorum* at different developmental stages, including egg, the first to fifth instars, and adults, using qRT-PCR. Our results showed that mRNAs of Al6 were more highly enriched at feeding stages (nymph and adult) than at the non-feeding stage (egg) (Fig. 3A), implying the important role of Al6 in plant feeding. Additionally, we detected the relative transcription of Al6 in different body parts, including salivary gland, head, thorax, abdomen, leg, and wing. qRT-PCR data suggested that AI6 in salivary glands had RNA changes ~10-fold greater than in other body parts such as the head, thorax, abdomen, leg, and wing (Fig. 3B). All of this evidence supported that Al6 was a potential salivary gland effector.

### Al6 acts as a secreted glutathione peroxidase

**Fig. 4.**
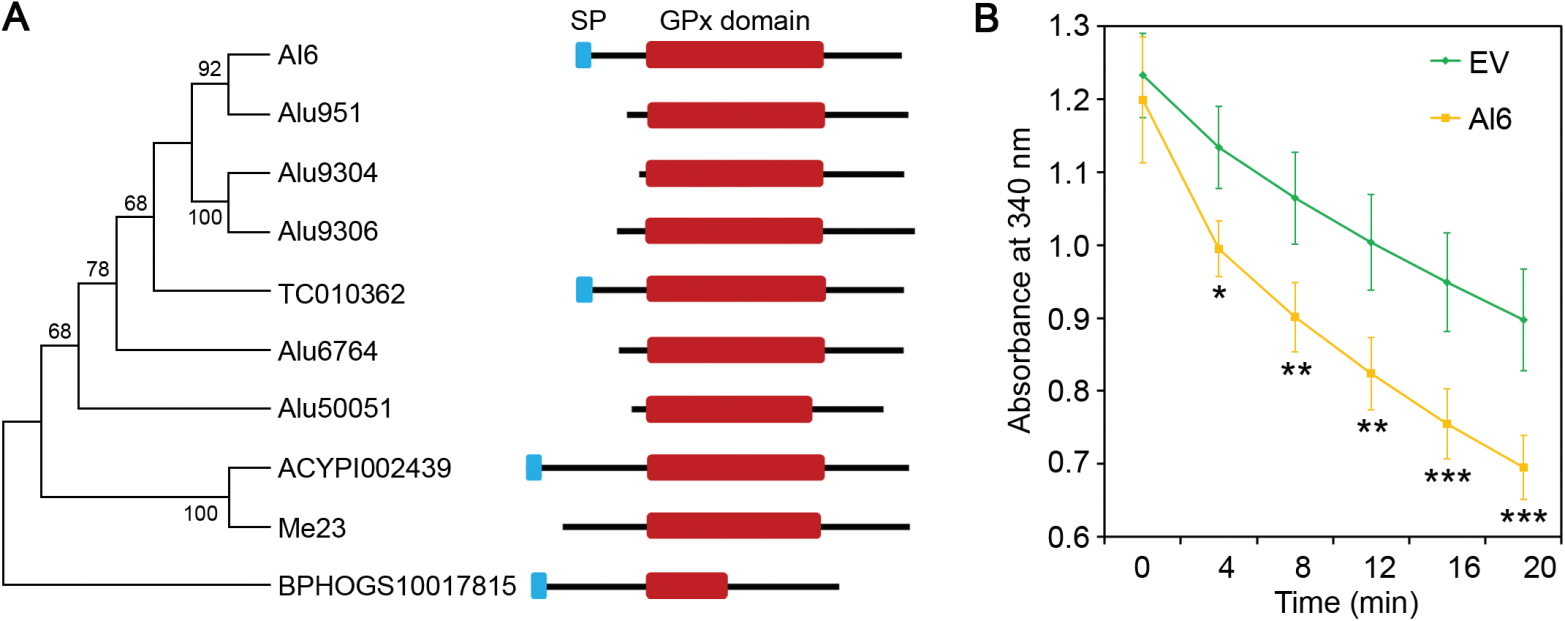
Al6 acts as a glutathione peroxidase. (A) Phylogenetic and domain structure analysis of GPx domain-containing proteins in *A. lucorum* and other insects. Alu951, Alu9304, Alu9306, Alu6764, and Alu50051 were the paralogs of Al6 in *A. lucorum*. Other homologs included TC010362 (*Tribolium castaneum*), ACYPI002439 (*Acyrthosiphon pisum*), Me23 (*Macrosiphum euphorbiae*), and BPHOGS10017815 (*Nilaparvata lugens*). (B) The enzymatic activity of Al6 measured by the absorbance of NADPH reduction at 340 nm. *His-Al6* and empty vector (*EV*) were expressed in *E. coli* strain BL21, and enzyme activities were assayed at 4, 8, 12, 16, and 20 min. Absorbance at 340 nm indicated decreased NADPH as representative of GPx activity. All the experiments were replicated three times and then analyzed with statistical methods (*P<0.01, **0.05<*P*<0.001, ***P<0.01, Student’s *t*-test). (This figure is available in color at *JXB* online.)

Further sequence analysis revealed that Al6 contained a conserved GPx domain (Fig. 4A; Supplementary Fig. S1), implying that Al6 may be an active enzyme for protection against hydroxyperoxides. Interestingly, several potential effectors with the GPx domain have been predicted in Hessian fly (*Mayetiola destructor*), pea aphid (*Acyrthosiphon pisum*), and potato aphid (*Macrosiphum euphorbiae*) (Chen *et al.*, 2008; Carolan *et al.*, 2011; Atamian *et al.*, 2013). Using conserved GPx domain sequences, homologs in the *A. lucorum* transcriptome and other sequenced herbivore genomes were characterized. A total of six proteins including Al6 were found to contain the GPx domain in *A. lucorum*, while only Al6 contained the N-terminal signal peptide (Fig. 4A). Meanwhile, the secreted proteins containing the GPx domain were also found in other insects, such as *Tribolium castaneum* and *Nilaparvata lugens* (Fig. 4A). Multiplex sequence alignment of Al6 and its homologs indicated that they shared high sequence similarity (Supplemetnary Fig. S1). This evidence supported the idea that secreted GPxs were present in several hemipteran and coleopteran species.

To further determine whether Al6 is a functional GPx, recombinant N-terminal His-tagged Al6 (His-Al6) and EV were expressed in *E. coli*. After purification of expressed protein, the enzymatic activity of Al6 was measured by detecting the reduction of NADPH substrate at an OD of 340 nm. A lower amount of substrate remaining indicates greater enzymatic activity. With the increase of reaction times, the NADPH substrate was reduced and the GPx activity of Al6 increased (Fig. 4B). Compared with the EV, enzymatic activity of Al6 was significantly stronger. Therefore, this result demonstrates that Al6 is an active GPx.

### Al6 suppresses PAMP-induced ROS

**Fig. 5.**
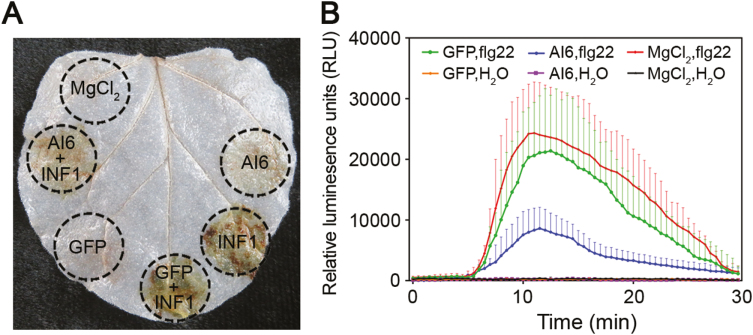
Al6 suppresses PAMP-induced ROS. (A) Al6 inhibits ROS accumulation induced by INF1. DAB staining was performed 2 d after infiltration with the leaves containing MgCl_2_, GFP, INF1, GFP/INF1, Al6/INFI, and Al6. (B) Al6 suppresses flg22-induced ROS production. Relative luminescence units (RLU) indicate the relative amounts of H_2_O_2_ production induced by 1 μM flg22. The results shown are representative of three independent experiments. Error bars indicate the SD. (This figure is available in color at *JXB* online.)

Based on the evidence that suppression of PTI induced by PAMPs was a common strategy employed by plant pathogen effectors, and the existence of the GPx domain in Al6, we attempted to explore the role of Al6 in ROS manipulation. INF1 was initially infiltrated in the same area of *N. benthamiana* leaves with expression of *Al6* after 24 hpi, and then DAB staining was used for analysis. DAB staining revealed that neither GFP nor Al6 induced ROS accumulation while INF1 triggered an oxidative burst; however, this ROS accumulation triggered by INF1 was significantly inhibited by co-expression with Al6. Thus, this evidence strongly supported that Al6 inhibited the ROS burst induced by INF1 (Fig. 5A). Further, we tested whether Al6 has an effect on the ROS production induced by another PAMP, flg22, from the flagellin of bacterial pathogens, which is the most commonly used ROS elicitor in large screens since this PAMP yields a strong and consistent ROS burst in *N. benthamiana* (Bos *et al.*, 2010). A luminol-based assay was performed to measure ROS production after flg22 treatment in the leaves of *N. benthamiana* transiently expressing *GFP-Al6* or the control *GFP*. Leaves with GFP expression showed a strong ROS burst in response to flg22; however, the accumulation of ROS was significantly reduced when *GFP-Al6* was co-expressed (Fig. 5B), showing that Al6 suppresses the ROS burst triggered by flg22. Taken together, our data showed that Al6 functions as a PTI inhibitor to suppress the PAMP-induced ROS burst.

### Al6 induces the hormone-related pathway

**Fig. 6.**
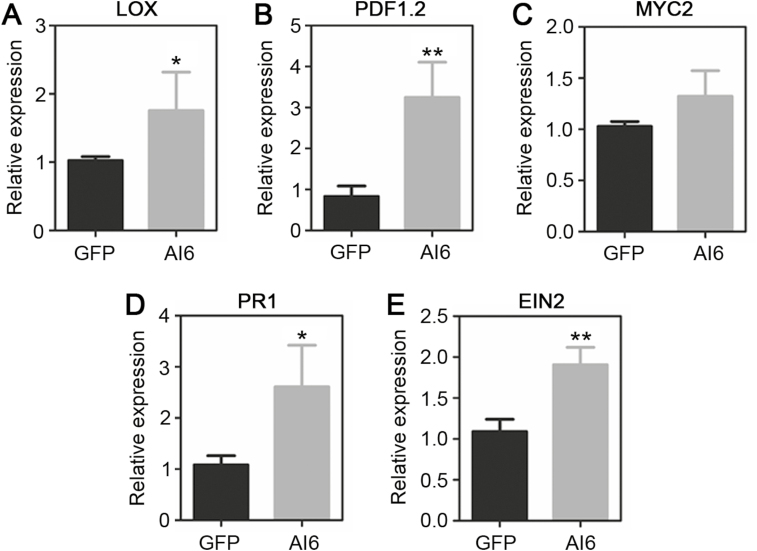
Al6 elicits the expression of key genes in hormone-related pathways. Expression levels of hormone-related genes following transient expression of *GFP* and *Al6* in *N. benthamiana* leaves. (A–C) JA pathway-related genes (*PDF1.2*, *LOX*, and *MYC2*). (D) The SA pathway-related gene *PR1*. (E) The ET pathway-related gene *EIN2*. Error bars represent the SD calculated from at least three repeats, and asterisks above the columns indicate significant differences compared with GFP (**P<0.01, *P<0.05, Student’s *t*-test).

Considering that hormones constitute an important class of signaling molecules in plant defense against biotic stress, including insect herbivores and microbial plant pathogens, we tested whether Al6 mediates hormone pathways. The key genes related to JA, SA, and ET were studied upon overexpression of *GFP* and *GFP-Al6* in *N. benthamiana* for 2 d. Our results showed that the RNA levels of the JA biosynthetic enzyme gene *LOX* (13-lipoxygenase) and the plant defensin gene *PDF1.2*, but not the myelocytomatosis protein gene *MYC2*, were up-regulated with an ~1- to 3.5-fold change after treatment with Al6 relative to GFP, implying that Al6 may trigger the JA pathway (Fig. 6A–C). Furthermore, the RNA levels of the SA-dependent genes *Pathogenesis-related 1* (*PR1*) and *Ethylene insensitive 2* (*EIN2*) in *Al6*-expressed leaves had ~2.5-fold changes (Fig. 6D, E). These data suggested that Al6 expression in plants activates the host hormone-related pathway.

### Al6 enhances plant susceptibility to biotic stress

**Fig. 7.**
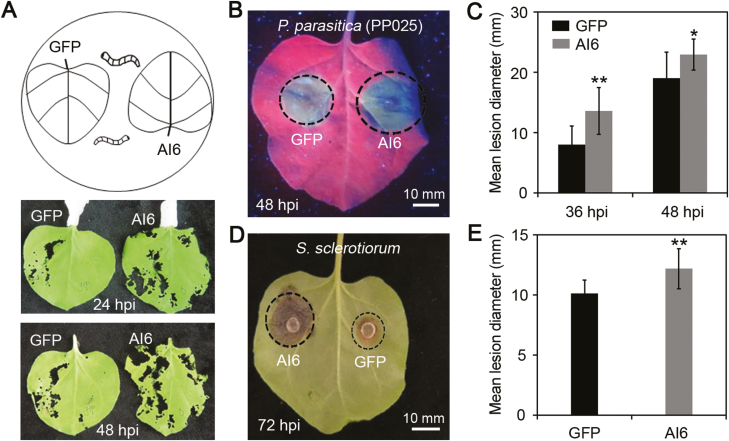
Al6 enhances plant susceptibility to biotic stress. (A) Al6 promotes cotton bollworm feeding on leaves with *Al6* expression. Eight second instar larvae of *H. armigera* were placed in the middle of leaves in which *GFP* and *Al6* were expressed, and photos were taken at 24 h and 48 h after treatment. All the samples were repeated at least three times. (B–E) Al6 decreases plant susceptibility to pathogens upon overexpression in *N. benthamiana*. (B and C) Leaves were infected with *P. parasitica*. Photographs were taken under UV light at 48 hpi (B), and the lesion diameter was measured at 36 and 48 hpi after treatment (C). (D and E) Leaves were infected with *S. sclerotiorum*. Disease symptoms were photographed under white light at 72 hpi (D); lesion diameter was measured and calculated from at least six independent biological repeats (E). Error bars represent the SD (**P<0.01, *P<0.05, Student’s *t*-test). (This figure is available in color at *JXB* online.)

Considering that Al6 manipulates the plant immunity by suppressing cell death and the ROS burst, it is reasonable to hypothesize that Al6 might change pest feeding behaviors and resistance to plant pathogens. To test this hypothesis, we designed an insect feeding assay with the cotton bollworm (*H. armigera*) which is a host of tobacco, by placing them in the middle area between two leaves individually expressing *GFP* and *Al6* (Fig. 7A). After 24 or 48 hpi, it was found that cotton bollworm were more prone to feed on the leaves expressing *Al6* than on the leaves expressing GFP (Fig. 7A). The result showed that the expression of *Al6* might promote herbivores such as *H. armigera* to feed on the plant.

Furthermore, we investigated whether Al6 had a potential role in enhancing host susceptibility to other biotic stresses, especially pathogens. In this study, we transiently overexpressed GFP and GFP–Al6 in *N. benthamiana*, and then challenged with the hemibiotrophic oomycete pathogen *P. parasitica* and the necrotrophic fungal pathogen *S. sclerotiorum*. *Phytophthora parasitica* and *S. sclerotiorum* were used for infection assay because they can cause easily recorded symptoms in the leave of *N. benthamiana.* We found that disease lesion diameters of *P. parasitica* or *S. sclerotiorum* were significantly larger in *Al6*-expressed leaves than in control leaves, showing that Al6 can promote the infection of either the oomycete pathogen *P. parasitica* or the fungal pathogen *S. sclerotiorum* (Fig. 7B–E), and indicating that Al6 negatively regulate the plant resistance to diseases. In conclusion, our results demonstrate that Al6 enhances plant susceptibility to biotic stress, including insect feeding and pathogen infection.

### Glutathione peroxidase activity is required for Al6 function

**Fig. 8.**
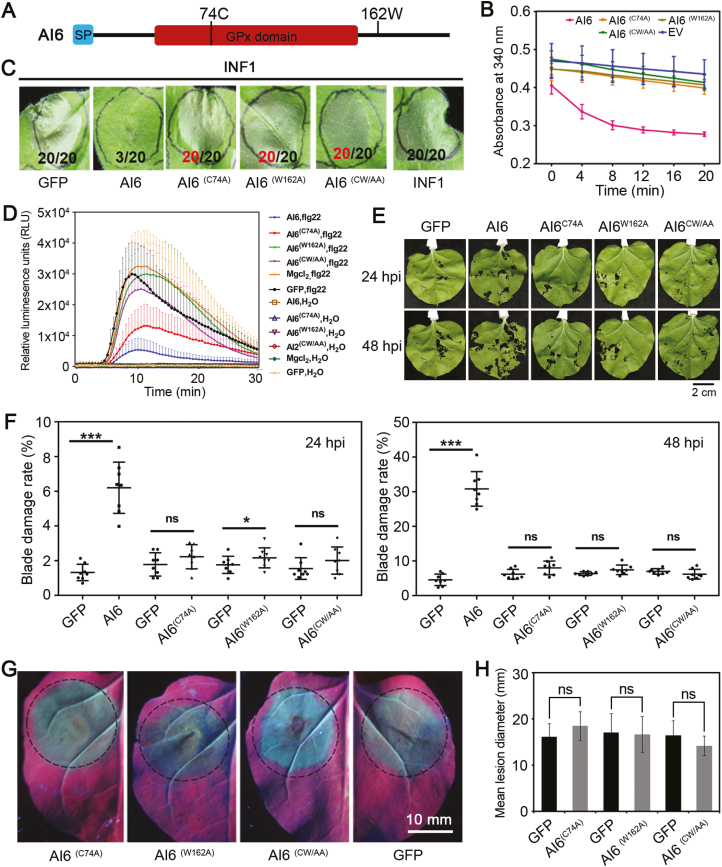
Glutathione peroxidase activity is required for Al6 function. (A) Schematic diagram showing the protein structure of Al6, together with its key sites. (B) Al6 mutants lose GPx enzyme activity. Al6, mutants, and EV were expressed in *E. coli* strain BL21, and enzyme activities were measured at an absorbance of 340 nm every 4 min. Wild-type Al6 with normal enzyme activity is the positive control, and EV serves as the negative control. (C) The mutants of Al6 lose capacity to suppress INF1-triggered cell death. *INF1* was expressed in *N. benthamiana* leaves 24 h after introduction of *Al6*, mutants (*Al6*^*C74A*^, *Al6*^*W162A*^, and *Al6*^*CW/AA*^), or *GFP* via agroinfiltration. The photograph was taken 3 d post-infiltration. (D) GPx activity of Al6 is essential for suppressing the ROS burst. ROS production was measured at 48 h after MgCl_2_ for *Al6* and mutants expressed in *N. benthamiana*. (E and F) Insect feeding assays of Al6 mutants and the wild type. Eight second instar larvae of *H. armigera* were placed in the middle of leaves in which *GFP* and *Al6* mutants (*Al6*^*C74A*^, *Al6*^*W162A*^, and *Al6*^*CW/AA*^) were expressed at 48 h, and photos were taken at 24 and 48 hpi. Each treatment was repeated eight times, and the damaged blade area and total blade area were calculated using ImageJ software. (G and H) Disease symptoms of leaves after *P. parasitica* infection were investigated. Half of an *N. benthamiana* leaf expressing *Al6*^*C74A*^, *Al6*^*W162A*^, or *Al6*^*CW/AA*^ and another half expressing GFP were infected with the pathogen *A. tumefaciens*. Photographs were taken under UV light at 48 hpi (G), and the lesion diameter was measured at 36 and 48 hpi (H). The data were calculated from three independent biological replicates using at least six leaves each. Error bars represent the SD, ns stands for significance (***P<0.001, **P<0.01, *P<0.05, Student’s *t*-test). (This figure is available in color at *JXB* online.)

To determine whether the GPx activity of Al6 was required for its function, we analyzed the active sites of this enzyme and selected potential residues for mutation. Previous evidence revealed that the 74th residue, a cysteine (C), was crucial for GPx catalytic activity, and the 162th residue, a tryptophan (W), was conserved among the members of the GPx protein families from insects, animals, and humans (Jiu *et al.*, 2015). Therefore, we created three mutants for Al6 by employing site-directed mutagenesis to replace the two key residues (C^74^ and W^162^) with alanine (Al6^C74A^, andAl6^W162A^), and mutating both of these residues (Al6^CW/AA^) (Fig. 8A). The enzyme activities of the three mutants and wild type of Al6 were determined. The results showed slightly lower curves of the three mutants (Al6^C74A^, Al6^W162A^, and Al6^CW/AA^), indicating that these mutants lost GPx activity. The NADPH substrate dramatically decreased over time for wild-type Al6, implying the active enzymatic activity of wild-type Al6 (Fig. 8B). Therefore, these results suggest that the residues C^74^ and W^162^ of Al6 are essential for the GPx activity.

For cell death-inhibiting assays of Al6 mutants, INF1 was infiltrated after the overexpression of *Al6* or mutants (Al6^C74A^, Al6^W162A,^ and Al6^CW/AA^) in the leaves of *N. benthamiana* to observe cell death symptoms. Figure 8C shows that the wild-type Al6 inhibited INF1-triggered cell death, but the three mutants did not, revealing that both key residues (C^74^ and W^162^) were required for Al6 to suppress cell death. We then tested whether the mutants of Al6 affect the ROS production induced by the PAMP flg22. A luminol-based assay was performed to measure ROS production in the wild type and mutant Al6 strains when they were transiently expressed in the leaves of *N. benthamiana*, using flg22 as a positive control and mock H_2_O as a negative control. Leaves with GFP expression or mock MgCl_2_ control showed a strong ROS burst in response to flg22, while leaves with Al6 expression showed significant inhibition of ROS burst. In comparison with Al6, GFP, or MgCl_2_, the abilities of mutant Al6^C74A^ and Al6^W162A^ to inhibit the flg22-induced ROS burst decreased, and even Al6^CW/AA^ lost this ability (Fig. 8D). Therefore, these results suggested that the GPx activity of Al6 is essential for suppressing the PAMP flg22-induced ROS burst. Further, we tested whether the mutants enhance host susceptibility by performing insect feeding assays with mutant Al6 lines. At 24 and 48 hpi, the larvae of cotton bollworm fed more on the leaves expressing Al6 than on those expressing GFP or Al6 mutants (Fig. 8E). The blade damage rate (damaged blade area/total blade area) was used to further verify this phenomenon, showing that Al6- expressing leaves were more severely damaged than mutant-expressing leaves (Al6^C74A^, Al6^W162A^, and Al6^CW/AA^) or GFP (Fig. 8F). So, our evidence revealed that Al6 mutants lost the ability to increase plant susceptibility to insects. Finally, we transiently overexpressed *GFP* and mutants (Al6^C74A^, Al6^W162A^, and Al6^CW/AA^) in *N. benthamiana*, and then challenged with *P. parasitica*. The disease lesion diameters of *P. parasitica* showed no significant difference in mutant expression leaves and the negative control at 48 hpi (Fig. 8G, H), indicating the Al6 mutants failed to promote pathogen infection. Taken together, these results suggested that Al6 suppressed INF1-induced cell death, inhibited PAMP-induced ROS, and enhanced host susceptibility to insect and pathogens in a manner dependent on the GPx activity of Al6.

## Discussion

In this study, a total of 1878 secreted proteins were identified from the *A. lucorum* transcriptome (Fig. 1), using a bioinformatics pipeline, and 172 secreted proteins were randomly selected from these proteins and transiently expressed in the leaves of *N. benthamiana* to perform cell death induction or suppression assays. No effector protein inducing plant cell death was obtained and one protein designated Al6 was characterized as inhibiting cell death induced by INF1 and the mouse BAX (Fig. 2). Further studies revealed that Al6 functioned as an active GPx enzyme to suppress the ROS burst induced by PAMPs such as INF1 or flg22, and thereby enhanced plant susceptibility to pathogens and insect feeding (Figs 4, 5). Two key residues (C^74^, W^162^) of Al6 were required for the GPx function. When these two residues were mutated, Al6 lost its ability to inhibit cell death and the ROS burst as well as resistance to biotic stresses (Fig. 8). Our finding provides the first evidence that *A. lucorum* employs effector proteins to promote performance on plants by suppressing the ROS signal pathway to manipulate plant defense reponses.

During insect feeding, plants perceive insect signals and trigger complex defense responses, such as the ROS burst, Ca^2+^ ion fluxes, the activation of plant MAPKs, and the induction of hormonal pathways (Acevedo *et al.*, 2015; Bigeard *et al.*, 2015). To enable successful feeding and infestation, insects deliver a series of effectors from salivary glands into their host cells to suppress plant defense responses and modulate herbivore–plant interactions (Hogenhout and Bos, 2011). Several insect herbivore effectors hijacking the plant signaling pathways have been described, for example the NlSEF1 protein from the brown planthopper, *Nilaparvata lugens* decreasing the cytosolic Ca^2+^ influx (Ye *et al.*, 2017) and HARP1 of *H. armigera* mediating the JA pathway (David *et al.*, 2016). Recently, a salivary ferritin, BtFer1 from whitefly *B. tabaci*, was found to be secreted into tomato (*Solanum lycopersicum*) for suppressing the ROS burst during feeding (Su *et al.*, 2019). So far, two candidate effectors, BtFer1 and Al6, have been functionally characterized from phloem-feeding insects to target the ROS pathway. As an iron storage protein, BtFer1 controls H_2_O_2_-generated oxidative signals by using ferroxidase to oxidize Fe^2+^ to the ferric state Fe^3+^ (Arosio *et al.*, 2009; Su *et al.*, 2019). Silencing BtFer1 expression increased H_2_O_2_ levels in plants following infestation by *B. tabaci* (Su *et al.*, 2019). On the other hand, Al6 utilizes GPx enzymatic activity to degrade toxic oxidation products produced when *A. lucorum* infests tobacco (Fig. 4). Therefore, these results suggest that piercing–sucking insects use multiple genes to suppress plant oxidative signaling induced by insect feeding.

This study investigated whether Al6 suppressed plant hormone signaling, a strong and fast defense mechanism triggered by various biotic stresses. Our data illustrated that the expression of *Al6* in plant cells triggered hormone-related pathways, as the results showed that defense marker genes in the JA, SA, and ET signaling pathways were increased upon the expression of *Al6* in *N. benthamiana* (Fig. 6). However, the study of BtFer1 suggested a potential link between the oxidative signaling and the JA signaling pathway, and both pathways were suppressed by BtFer1 at the same time (Su *et al.*, 2019). A plausible explanation for these contradictory results is that *B. tabaci* BtFer1 and *A. lucorum* Al6 employ different tactics to manipulate plant immune responses. Our evidence implies that PAMP-induced ROS accumulation and hormone pathways may be independent, and effector molecules such as Al6 might have difficulty targeting both pathways simultaneously (Bigeard *et al.*, 2015). Our result was also supported by the report that the aphid cell death-inducing effector Mp10 activated JA and SA signaling pathways (Bos *et al.*, 2010; Rodriguez *et al.*, 2014). NlSEF1 protein from the brown planthopper regulates the levels of Ca^2+^ and H_2_O_2_, but not JA and SA signaling in rice (Ye *et al.*, 2017). Overall, this research further broadens the study of insect salivary gland effectors and provides additional information on the working mechnisms of insect effectors interfering with the plant immune system.

The effects of Al6 on plant performance in response to insect behavior and pathogens were studied by insect feeding and pathogen infection assays. We found that overexpression of *Al6* promoted the feeding of the cotton bollworm *H. armigera* on the leaves (Fig. 7A), implying that Al6 changed herbivore feeding behaviors by suppressing plant immunity. As reported earlier, aphids showed a significant preference for *N. benthamiana* expressing the effector gene *Mp55* from *Myzus persicae* compared with controls (Elzinga *et al.*, 2014). Several other studies also indicated that insect effectors regulate their hosts to promote feeding and reproduction (Atamian *et al.*, 2013; Guiguet *et al.*, 2016). Additionally, our result suggested that Al6 promoted infection by the hemibiotrophic plant pathogen *P. parasitica* and the necrotrophic plant pathogen *S. sclerotiorum* in *N. benthamiana* (Fig. 7B–E). Our data were consistent with aphid Mp10 decreasing susceptibility to the hemibiotrophic plant pathogen *Phytophthora capsici* (Rodriguez *et al.*, 2014). Using three GPx loss-of-function mutants, this research also demonstrated that destroying GPx activity of Al6 restored the resistance to the plant pathogen *P. parasitica* (Fig. 8G, H). Therefore, these studies indicate that phloem-feeding insects injecting salivary gland effectors into the host cell to interfere with plant susceptibility to insects and pathogens is a common tactic.

### Conclusions

A secreted salivary gland candidate effector, Al6, with GPx enzyme activity was identified from *A. lucorum*. Upon overexpression in the leaves of *N. benthamiana*, Al6 suppressed the ROS induced by the PAMPs INF1 and flg22, and inhibited PTI-induced cell death. The expression of *Al6* in plant cells activated hormone-related pathways, such as JA, SA, and ET. By manipulating the PTI signaling pathways, Al6 changes the feeding behavior of herbivores such as *H. armigera* and promotes the infection of the hemibiotrophic plant pathogen *P. parasitica* and the necrotrophic plant pathogen *S. sclerotiorum*. Overall, this study provides evidence for a secreted GPx protein from the salivary gland of *A. lucorum* targeting host PTI-induced ROS burst and cell death, and offers a new insight into the molecular mechanism underlying insect–plant interactions.

## Supplementary data

Supplementary data are available at *JXB* online.

Fig. S1. Multiple sequence alignment of GPx domain-containing proteins.

Table S1. Summary of the qRT-PCR primers used in this study.

eraa015_suppl_supplementary_figure_S1_table_S1

## Abbreviations:

DAB: 3,3′-diaminobenzidine
ET: ethylene
ETI: effector-triggered immunity
EV: empty vector
GPx: glutathione peroxidase
hpi: hours post-infiltration or inoculation
JA: jasmonic acid
JAZ: JASMONATE-ZIM-DOMAIN
MAPK: mitogen-activated protein kinase
OD: optical density
PAMP: pathogen-associated molecular pattern
PTI: pattern-triggered immunity
ROS: reactive oxygen species
SA: salicylic acid
