# Supplementary material for: The mirid bug Apolygus lucorum deploys a glutathione peroxidase as a candidate effector to enhance plant susceptibility
Source: J Exp Bot. 2020 Jan 17;71(9):2701–12. doi: 10.1093/jxb/eraa015 (PMC7210764; doi:10.1093/jxb/eraa015)
Supplement: eraa015_suppl_supplementary_figure_S1_table_S1 [file eraa015_suppl_supplementary_figure_s1_table_s1.pdf]

Table S1：Summary of the qRT-PCR primers used in this study.

**Name**	**Sequence (from 5' to 3')**	**Usage**
Pbin-AI6-F	GACGAGCTGTACAAGGGTACCATGG TCAGATCATTTGCAGTTGCAGC	Expression in *N.benthamiana*
Pbin-AI6-R	GCGGACTCTAGTTCATCTAGATTACT GTTCCAACAGCTTTTTTA	Expression in *N.benthamiana*
ALActin RT-F	TCTTGCGGTATCCACGAAAC	*Apolygus lucorum* beta-actin Mrna , used for real time PCR assay
ALActinRT- R	GAGGGCGGTGATTTCCTTCT	*Apolygus lucorum* beta-actin Mrna , used for real time PCR assay
AI6-RT-F	AGAGTGCACAGTCGGGATGGT	qRT-PCR primer for AI6 transcription level
AI6-RT-R	TGGGGTGGTTGGTGCATGTCG	qRT-PCR primer for AI6 transcription level
NbEF1a-RT-F	TATGATTACTGGTACCTCCC	Internal primers of *N. benthamiana EF1α*, used for real time PCR assay
NbEF1a-RT-R	ACCTAGCCTTGGAATACTTG	Internal primers of *N. benthamiana EF1α,* used for real time PCR assay
LOX-RT-F	AAAACCTATGCCTCAAGAAC	qRT-PCR primer for JA pathway marker gene
LOX-RT-R	ACTGCTGCATAGGCTTTGG	qRT-PCR primer for JA pathway marker gene
PDF1.2-RT-F	CTTCAAGCAAAGCTGCAGCCAAAG	qRT-PCR primer for JA pathway marker gene
PDF1.2-RT-R	CTATGCACTAAGCCATGTGTGTTTG	qRT-PCR primer for JA pathway marker gene
MYC2-F	GAAGCGGATAGTAGTAGAGTT	qRT-PCR primer for JA pathway marker gene
MYC2-R	TTTCTCCCTCCTTTGTCT	qRT-PCR primer for JA pathway marker gene
PR1-RT-F	TGAGATGTGGGTCGATGAGA	qRT-PCR primer for SA pathway marker gene
PR1-RT-R	CGAGTTACGCCAAACCACTT	qRT-PCR primer for SA pathway marker gene
EIN2-RT-F	TCACTCGGAAGAGGAAGC	qRT-PCR primer for ET pathway marker gene
EIN2-RT-R	TGCGGACATTGAAGACAC	qRT-PCR primer for ET pathway marker gene
PET32a-AI6-F	GACAAGGCCATGGCTGATATCATGG TCAGATCATTTGCAGTTGCAGC	Expression in *E.coil* stain BL21
PET32a-AI6-R	GGTGGTGGTGGTGGTGCTCGAGCTG TTCCAACAGCTTTTTTA	Expression in *E.coil* stain BL21
CA-F1c	GCCAGTTTGGCTGGACTA	Primer for mutant construction
CA-R1b	TAGTCCAGCCAAACTGGC	Primer for mutant construction
WA-F3c	GCTATCAAAGCGAATTTCACC	Primer for mutant construction
WA-R3b	GGTGAAATTCGCTTTGATAGC	Primer for mutant construction

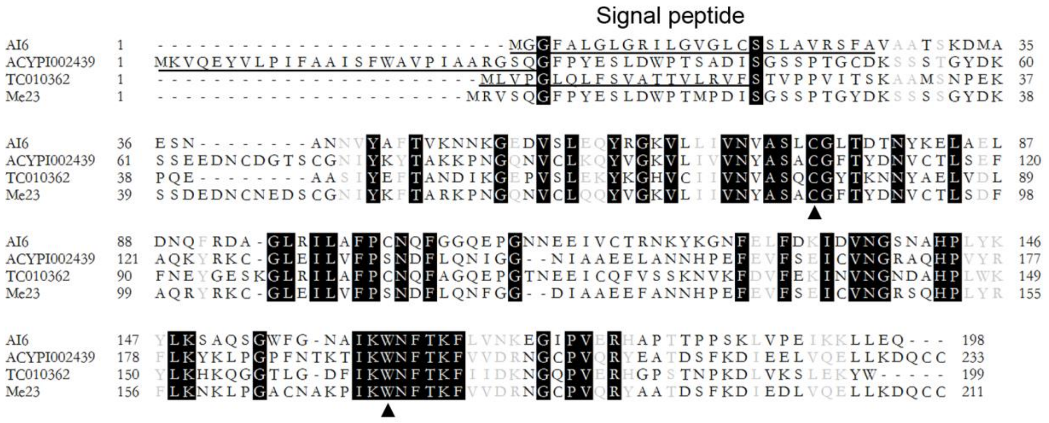

Figure S1. Multiple sequence alignment of GPx domain-containing proteins. The identical amino acids in the alignment are highlighted in black. The known functional amino acid residues engaged in the formation of the catalytic centre are marked with filled arrowheads.
